# Targeted Sequencing and Meta-Analysis of Preterm Birth

**DOI:** 10.1371/journal.pone.0155021

**Published:** 2016-05-10

**Authors:** Alper Uzun, Jessica Schuster, Bethany McGonnigal, Christoph Schorl, Andrew Dewan, James Padbury

**Affiliations:** 1 Department of Pediatrics, Women & Infants Hospital of Rhode Island, Providence, Rhode Island, United States of America; 2 Brown Alpert Medical School, Providence, Rhode Island, United States of America; 3 Molecular Biology, Cell Biology & Biochemistry, Brown University, Providence, Rhode Island, United States of America; 4 Department of Epidemiology and Public Health, Yale University, New Haven, Connecticut, United States of America; 5 Center for Computational Molecular Biology, Brown University, Providence, Rhode Island, United States of America; Center of Genomic & Post Genomics, ITALY

## Abstract

Understanding the genetic contribution(s) to the risk of preterm birth may lead to the development of interventions for treatment, prediction and prevention. Twin studies suggest heritability of preterm birth is 36–40%. Large epidemiological analyses support a primary maternal origin for recurrence of preterm birth, with little effect of paternal or fetal genetic factors. We exploited an “extreme phenotype” of preterm birth to leverage the likelihood of genetic discovery. We compared variants identified by targeted sequencing of women with 2–3 generations of preterm birth with term controls without history of preterm birth. We used a meta-genomic, bi-clustering algorithm to identify gene sets coordinately associated with preterm birth. We identified 33 genes including 217 variants from 5 modules that were significantly different between cases and controls. The most frequently identified and connected genes in the exome library were IGF1, ATM and IQGAP2. Likewise, SOS1, RAF1 and AKT3 were most frequent in the haplotype library. Additionally, SERPINB8, AZU1 and WASF3 showed significant differences in abundance of variants in the univariate comparison of cases and controls. The biological processes impacted by these gene sets included: cell motility, migration and locomotion; response to glucocorticoid stimulus; signal transduction; metabolic regulation and control of apoptosis.

## Introduction

Despite significant advances in the care of pregnant mothers and infants, preterm birth remains a leading cause of newborn morbidity, mortality and hospitalization in the first year of life in the United States [1]. In developed countries 70% of infant mortality is secondary to preterm birth (birth before 37 completed weeks of gestation). The rate of preterm birth varies in different societies and in different ethnic groups from 3.8% in Eastern Asia to rates reaching close to 17% in disadvantaged African American groups [2, 3]. Neonatal morbidity and mortality after preterm birth are inversely related to gestational length. Survivors are at increased risk of cerebral palsy, intellectual disabilities, respiratory problems and other long term conditions[4]. Moreover, despite numerous attempts at intervention, the incidence of prematurity has shown minimal improvement over the last two decades [2]. The risk factors associated with prematurity are many including: adverse sociodemographic status, race/ethnicity, infection, stress, trauma and prior history of a premature birth [4–10]. The leading etiology is idiopathic. A large number of clinical/epidemiologic studies have examined the individual and collective contribution of each of these factors. A family history of preterm birth and inter-pregnancy interval of <18 months also increase the risk of prematurity [9].

A precise estimate of the contribution(s) of genetic factors to preterm birth has been difficult to achieve [11–17]. Twin studies suggest heritability is 36–40%, however differences in gestational age used and other details cloud the precision of those estimates [18, 19]. A history of a previous preterm birth is one of the best predictors of a subsequent preterm delivery. Mothers who were preterm themselves or who have a first order relative with preterm birth have an increased risk for delivering prematurely. These observations support the importance of genetic factors in preterm birth [13, 20, 21]. Large epidemiological studies drawn from population based analyses in Sweden and Denmark support a maternal origin for the genetic contribution(s) to risk of preterm birth, with little contribution by paternal or fetal genetic factors [17, 22–24].

Attempts to identify the genetic contributions to the risk of preterm birth have been pursued widely [13–17, 25, 26]. Studies have focused on genomic and proteomic approaches to the mechanism(s) of preterm labor. Polymorphic changes in the protein coding regions, regulatory and intronic sequences of specific genes have been described. In most of these studies, candidate genes or proteins involved in inflammatory reactivity or uterine contractility have been investigated [13–18, 25–37]. The results suggest that alteration in the expression of these proteins interacts with infection and/or other environmental influences associated with preterm birth. The results however, do not provide insight into the causes of prematurity in the absence of early inflammation or infection. Moreover, while interventions directed at infection or inflammation have been successful in experimental models they have largely been unsuccessful in treatment or prevention of preterm birth in humans [38]. Thus, there is abundant information that demonstrates important genetic contribution(s) to the risk of preterm birth and further suggests that preterm birth is a complex, polygenic disorder that entails activation and/or suppression of a host of genes [4]. In addition, linkage analyses have been limited because large pedigrees with a family history of preterm birth are not widely available, however one such study has been reported [39]. In spite of the data suggesting an association between genetics and PTB, there is a gap in our knowledge of the precise genetic contributions and whether they are discrete or multifactorial.

We have developed an alternative approach to identify a more manageable set of genes for preterm birth which incorporates some elements of the discovery in genome wide investigations. We previously used a bioinformatics approach for mining published literature and screening publicly available high-throughput databases to develop a validated collection of genes with *a priori* connection to preterm birth [40]. We used gene set enrichment analysis (GSEA) of this refined gene set to analyze a large genome wide association study to identify the contribution(s) of individual biological pathways to the genetic architecture of preterm birth [40, 41]. We identified important genes and networks associated with preterm birth. In order to identify the variants underlying these associations, we targeted the exons, flanking sequence and splice sites of the 329 genes and 132 haplotype blocks that we showed were associated with preterm birth [41]. We were as interested in variants that were associated with increased risk for preterm birth as we were with variants that were associated with reduced risk. We exploited an “extreme phenotype” of preterm birth to leverage the likelihood of genetic discovery by concentrating our enrollment on patients with a prior history of preterm birth. We compared variants identified in women with 2–3 generations of preterm birth with term controls without history of preterm birth. We used a meta-analytic, bi-clustering algorithm to identify gene sets coordinately associated with preterm birth. We identified 33 genes including 217 variants from 5 modules significantly different between cases and controls. The biological processes impacted by these gene sets included: cell motility, migration and locomotion; response to glucocorticoid stimulus; signal transduction; metabolic regulation and control of apoptosis.

## Results

### Library Design and Univariate Sequence Analysis

**Fig 1:**
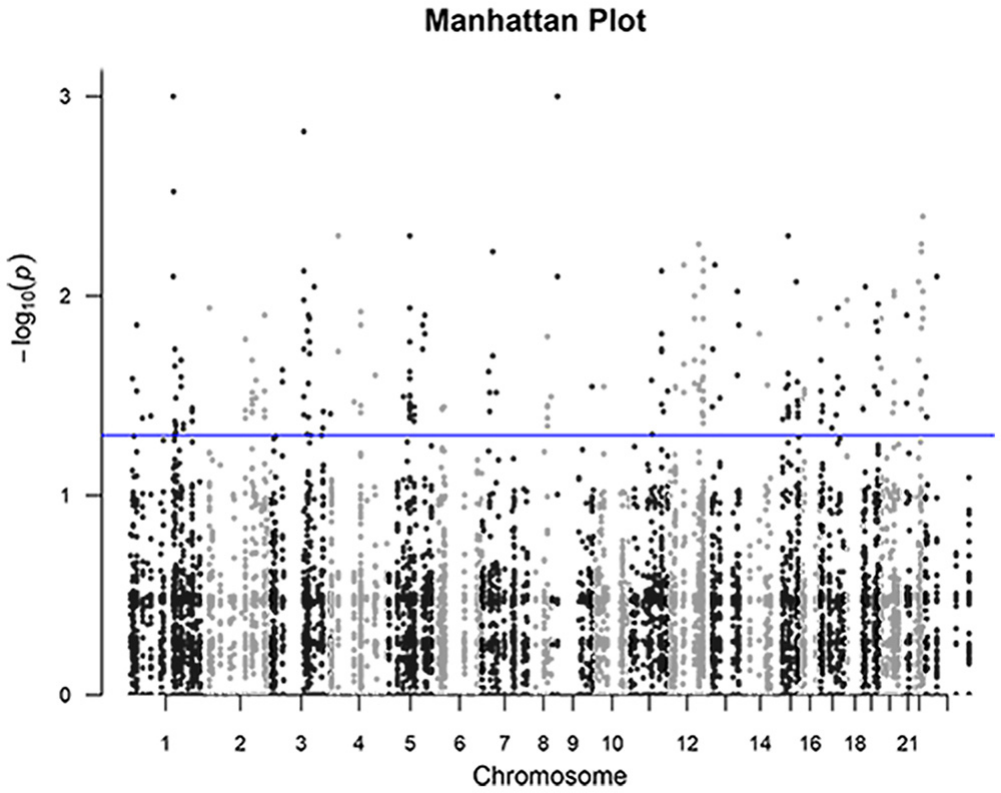
Manhattan Plot of Significant Variants. The 13,000 variants from the targeted exome library and 11,000 variants from the haplotype block library were compared for difference in abundance in the cases versus the controls. The figure shows a Manhattan plot of all variants across 22 autosomes with the vertical axis being the -logP value from the statistical test for association, with the threshold line (-logP 1.3) indicating p-value of 0.05. There were 205 and 168 variants that significantly differed in abundance in cases versus controls from the exome and haplotype block libraries respectively.

Sequencing was carried out on 32 women with 2 or 3 generations of preterm birth and 16 controls. We targeted the exons, flanking sequence and splice sites of the 329 genes and 132 haplotype blocks that we had previously shown were highly associated with preterm birth [41]. We identified over 13,000 variants in the targeted exome library and 11,000 variants in the haplotype block library [41]. Using the univariate analysis strategy discussed in the Methods, we identified 205 and 168 variants that were significantly different in abundance between cases and controls from the exome and haplotype block libraries at p<0.05, respectively. These variants and their associated genes are shown in S1 and S2 Tables. Fig 1 shows a Manhattan plot for these combined results with a threshold at -logP 1.3.

### Meta-analysis

**Fig 2:**
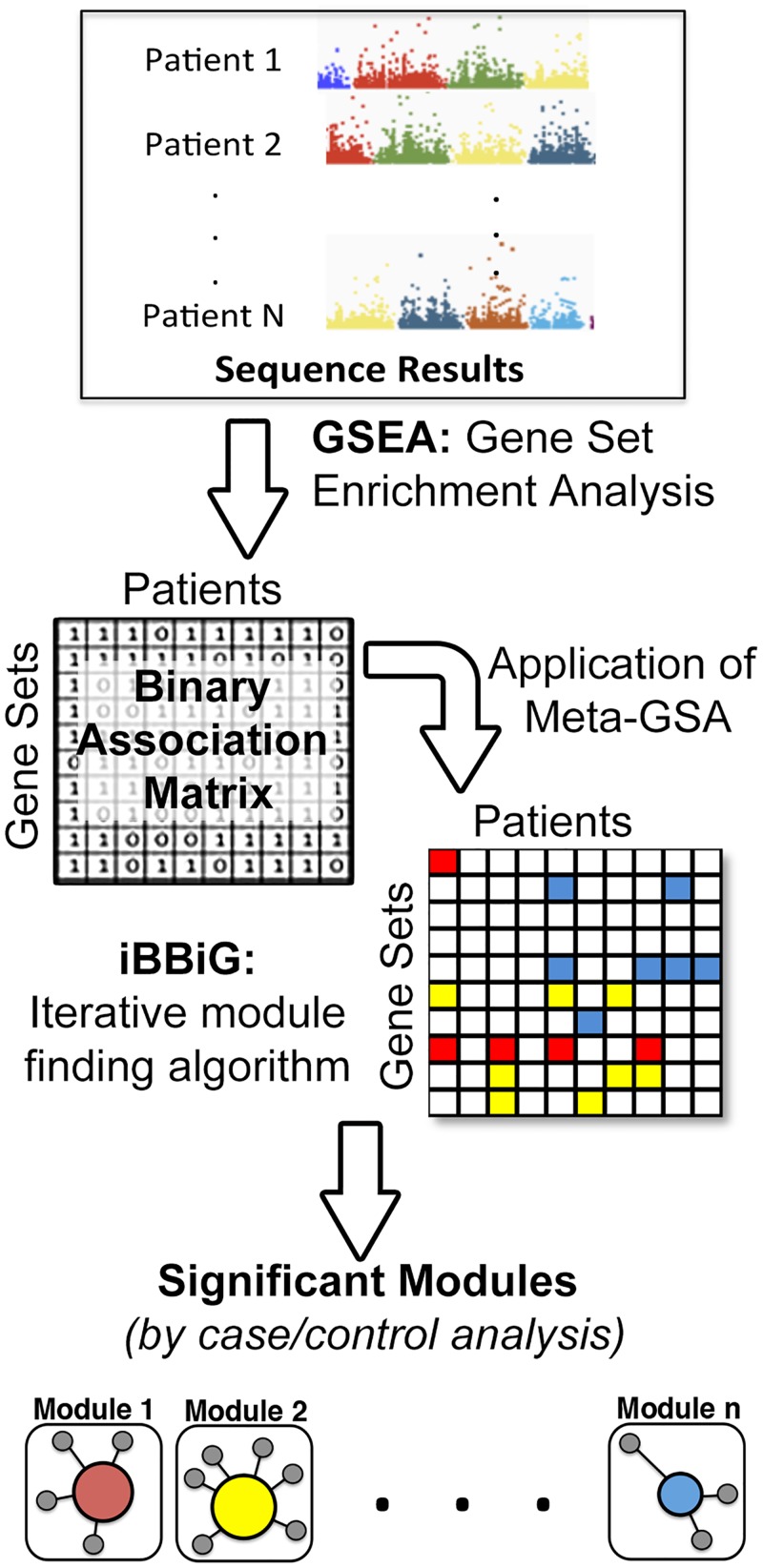
Meta-analysis and analytical pipeline: The genes harboring variants in each patient were analyzed by gene set enrichment using the MSig database C2 collection of gene sets [43]. The significant gene sets for each patient were combined into a binary association matrix. The iBBiG algorithm extracts modules of gene sets and patient subsets from the data matrix. The modules are represented by different colors. Fisher’s exact test was used to identify modules with significant differences in the number of cases and controls.

The genes containing variants that showed significant differences between cases and controls were examined for their association with networks and biological pathways using GSEA. We analyzed genes whose variants differed from controls with a p-value <0.1. We ran GSEA independently for each of the 48 patients. The significant gene sets from the GSEA of each patient were then compared by adapting a newly described meta-analytic approach known as iterative binary bi-clustering (iBBiG) [42]. The iBBiG algorithm identifies “modules” of gene sets and patient subsets from binary data [42]. Our analytical pipeline is illustrated in Fig 2.

**Fig 3:**
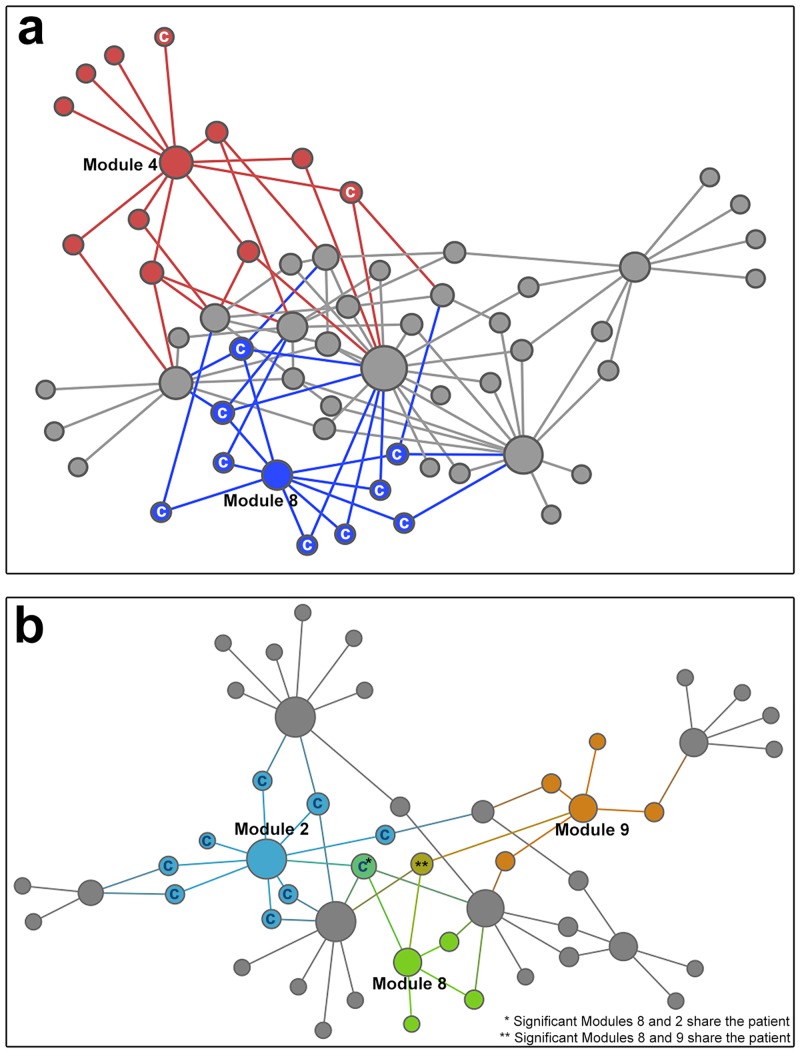
Network analysis of cases and controls. Cases are labeled by letter “c” in the significant modules. **(A)** Network of all modules of the exome library and patients, with the patients from significant Modules 4 and 8 highlighted in red and blue, respectively. **(3B)** Network of all modules of the haplotype block library and patients, with the patients from significant Modules 2, 8 and 9 highlighted in light blue, green and orange, respectively.

For each module we analyzed the patient subsets by comparing the number of cases versus the number of controls. These results are summarized in Table 1, which lists the module number, the numbers of cases, the numbers of controls, and the p-value (Fisher’s exact test). This analysis of the exome library identified 2 modules, for which there were significant differences in number of cases and controls in the patient subsets. For the haplotype library 3 significant modules were identified. Fig 3A and 3B shows a network of all of the modules and the patients assigned to each module from the exome and haplotype libraries. For the exome library, Module 8 (blue) contained significantly more cases than controls and Module 4 (red) contained significantly more controls than cases. For the haplotype library, Module 2 (light blue) contained significantly more cases than controls and Module 8 (green) and Module 9 (orange) contained significantly more controls than cases. Not surprisingly, patient subsets overlapped between modules. However for the exome library, the significant modules were represented by discrete patients. In the haplotype library two patients were shared among the 3 significant modules.

**Table 1:** The number of case and control patients and Fisher’s Exact p-value for each of the significant modules from the two targeted sequencing libraries.

Libraries	Modules	Cases	Controls	p-value
**Exome Library**	M4	2	9	0.0002
	M8	9	0	0.0200
**Haplotype Library**	H2	9	0	0.0200
	H8	1	4	0.0300
	H9	0	5	0.0025

**Fig 4:**
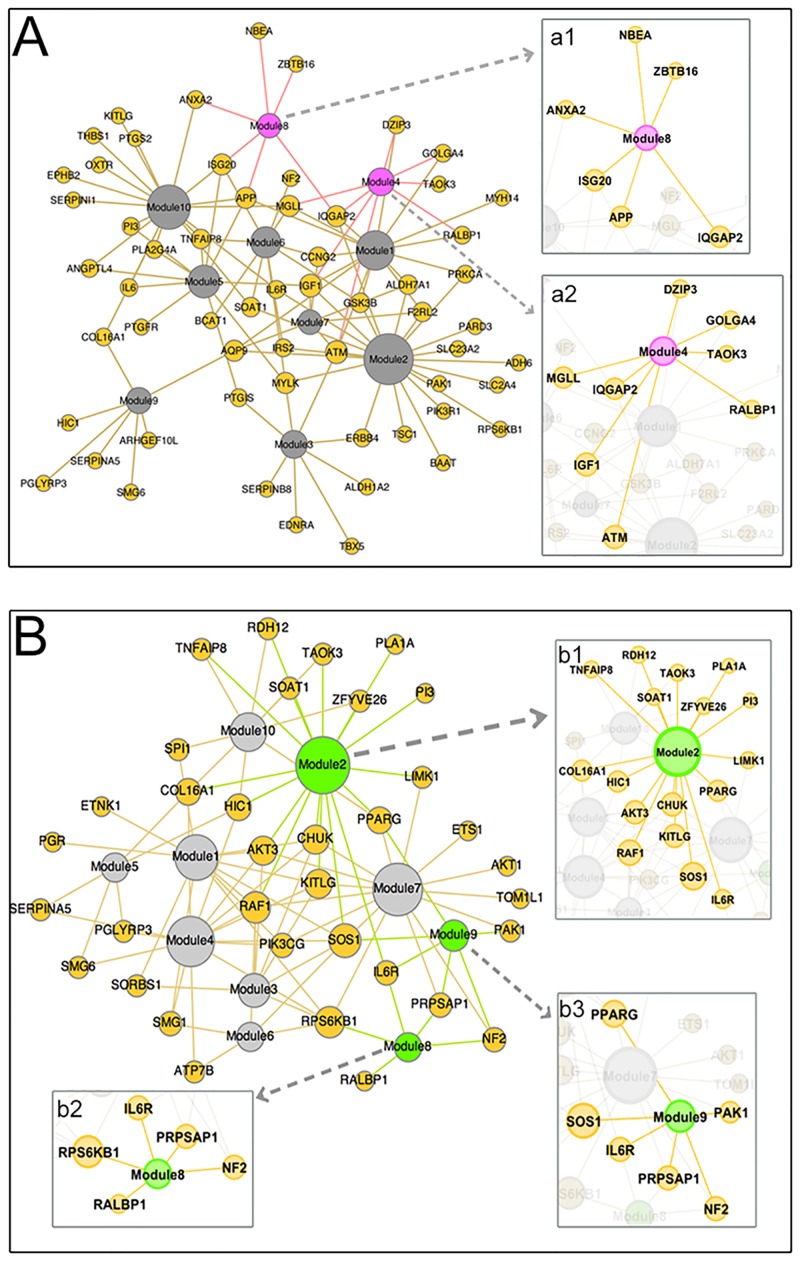
Network of modules and their gene sets. **(A)** Network output showing all 10 modules from the exome library and the genes contained in each module. The two significant modules are displayed as insets. Inset **a1** and **a2** display the genes of E8 and E4 respectively. **(B)** Network output showing all 10 modules from the haplotype block library and the genes contained in each module. Insets **b1, b2 and b3** show the genes of H2, H9 and H3 respectively.

In addition to patient subsets, each module contained gene sets. We extracted the genes from each gene set. We found significant overlap when analyzing these modules. Several gene sets were included in more than one module and there were multiple genes within each gene set that were shared among modules. This overlap is displayed for the exome library and the haplotype block library in Fig 4A and 4B. The significant modules and associated genes are displayed as Insets of Fig 4A and 4B. Inset **a1** shows the 6 genes in Module 8 and inset **a2** shows the 8 genes in Module 4. Inset **b1** shows the 17 genes in Module 2, **b2** shows the 5 genes in Module 8 and **b3** shows the 6 genes in Module 9. Table 2 lists these individual genes, their genomic location, and the five significant modules to which they belong.

**Table 2:** Gene names, IDs and chromosome numbers identified in the significant modules from both targeted sequencing libraries.

Gene	HGNC ID	Chr	Modules
DZIP3	30938	3	E4
GOLGA4	4427	3	E4
TAOK3	18133	12	E4, H2
RALBP1	9841	18	E4, H8
IGF1	5464	12	E4
ATM	795	11	E4
MGLL	17038	3	E4
IQGAP2	6111	5	E4,E8
NBEA	7648	13	E8
ZBTB16	12930	11	E8
APP	620	21	E8
ISG20	6130	15	E8
ANXA2	537	15	E8
TNFAIP8	17260	5	H2
RDH12	19977	14	H2
SOAT1	11177	1	H2
PLA1A	17661	3	H2
PI3	8947	20	H2
LIMK1	6613	7	H2
PPARG	9236	3	H2, H9
ZFYVE26	20761	14	H2
SOS1	11187	2	H2, H9
KITLG	6343	12	H2
CHUK	1974	10	H2
RAF1	9829	3	H2
AKT3	393	1	H2
HIC1	4909	17	H2
COL16A1	2193	1	H2
IL6R	6019	1	H2, H8, H9
PRPSAP1	9466	17	H8, H9
NF2	7773	22	H8, H9
RPS6KB1	10436	17	H8
PAK1	8590	11	H9

**E4 =** Exome Library Module 4, **E8 =** Exome Library, Module 8,

**H2 =** Haplotype Library, Module 2, **H8 =** Haplotype Library, Module 8, **H9 =** Haplotype Library, Module 9

The most highly connected genes in the exome library, IGF1, ATM and IQGAP2, were identified in 4–5 modules, Fig 4A. Similarly, from the haplotype block library, SOS1, RAF1 and AKT3 were identified in 5–6 modules, Fig 4B.

We recognize that some of the individual variants we found during the initial univariate testing might be important and not identified in the meta-analysis. Table 3 shows the variants in SERPINB8, AZU1 and WASF3 that were significantly different in cases and controls (p <0.05) with predicted deleterious effects according to PolyPhen 2 HDIV.

**Table 3:** Significant variants from the Exome library with annotations.

Gene	HGNC id	Chr	Function	dbSNP 138	Polyphen 2HDIV	p-value
SERPINB8	8952	18	exonic	rs3826616	0.998	0.036
AZU1	913	19	exonic	rs28626600	0.995	0.037
WASF3	12734	13	exonic	rs17084492	0.968	0.036

### Gene Ontology Analysis

**Fig 5:**
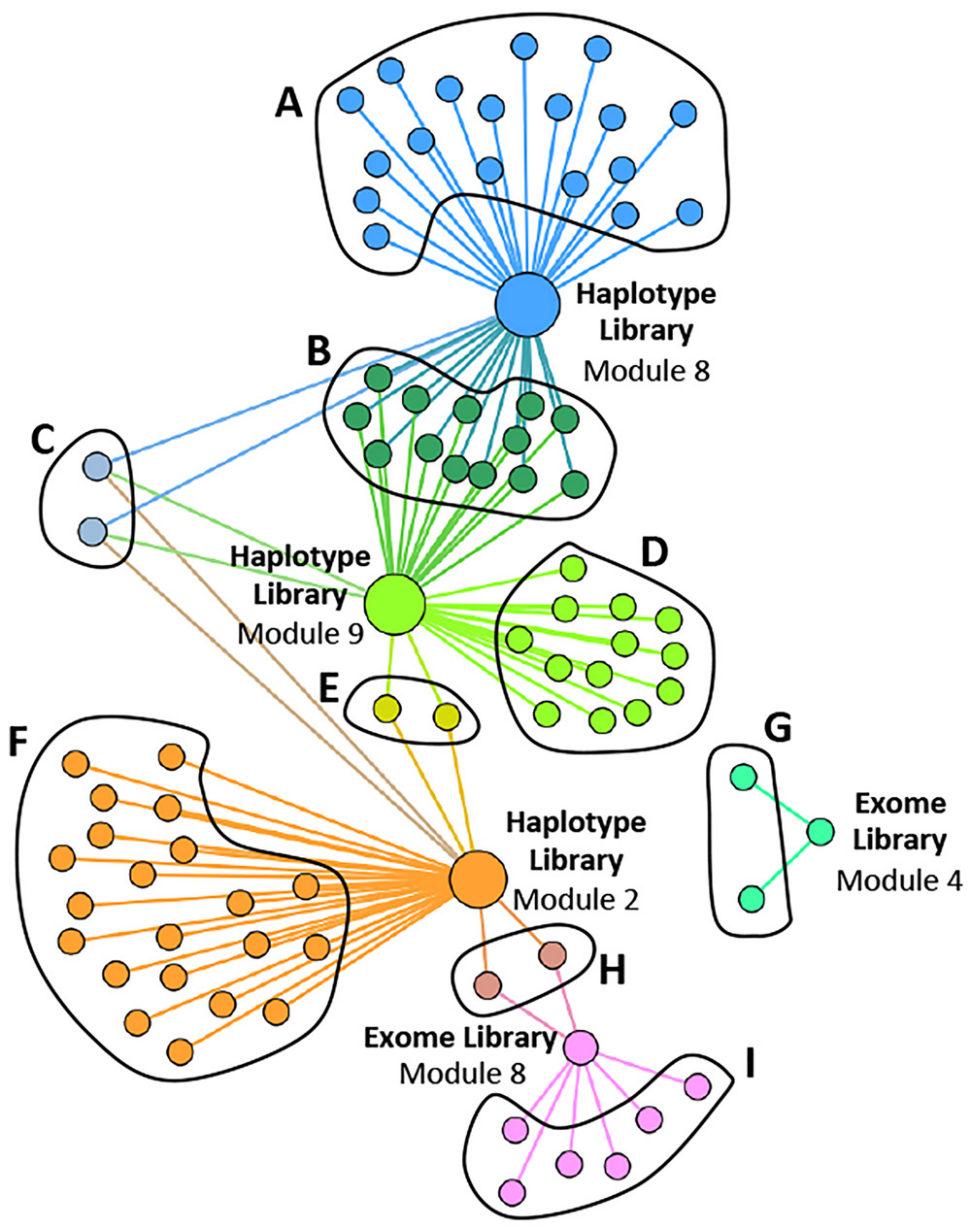
Ontology groups. Diagram showing the clusters of terms from the Gene Ontology analysis for biological processes related to preterm birth. Gene Ontology Database terms for biological processes shown in clusters A thru I are detailed in S3 Table.

We performed gene ontology analysis to identify the biological processes for the genes belonging to the significant modules [44]. A total of 80 groups of biological processes were identified which segregated into 9 individual and overlapping clusters, S3 Table. Fig 5 shows these individual and shared ontology groups. The most abundant association was with mechanisms regulating programmed cell death. Likewise, control of cell motility, migration and cell cycle regulation were associated with several of the most highly connected genes in Module 4 of the exome library. Metabolic processes, phosphate and lipid metabolism, protein phosphorylation and various forms of signal transduction were common biological functions attributed to the other most highly connected genes from the exome library. Similarly, the results from the gene ontology analysis of the haplotype library showed a high degree of association with cellular metabolism, signal transduction and nucleic acid metabolism. Regulation of immune cell system development, responses to glucocorticoid signaling, signal transduction pathways in immune regulatory cells and regulation of smooth muscle cell proliferation were associated with the genes identified in the haplotype library.

## Discussion

We are striving to identify the genetic basis for preterm birth. Our prior pathway analysis supports our strategy to look for variants in shared networks or pathways of genes that contribute to risk or resilience [41]. In order to increase the likelihood of discovery, we leveraged genetic risk by concentrating our enrollment on patients with a prior history of preterm birth. We compared variants identified in women with 2–3 generations of preterm birth with term controls without history of preterm birth. We performed targeted sequencing of the haplotype blocks surrounding previously identified significant variants from a GWAS for preterm birth [41]. We also performed targeted sequencing of the exons and flanking sequences of the genes nearest these variants. We then filtered the resulting 25,000 variants for those which were significantly different between preterm birth cases and matched controls. We adapted a new meta-analysis to identify modules of gene sets that were increased in abundance in cases or the controls. Several gene sets were included in more than one module and there were multiple genes within each gene set that were shared among modules. The most frequently identified and connected genes in the exome library were IGF1, ATM and IQGAP2. Likewise, SOS1, RAF1 and AKT3 were most frequent in the haplotype library. Additionally, SERPINB8, AZU1 and WASF3 showed significant differences in abundance of variants in the univariate comparison of cases and controls. IGFI has been previously implicated in preterm birth in association with variants in coagulation and inflammation pathway genes [45]. IGF1 has also been implicated through reduced expression in the placenta from preterm birth compared to controls [46]. The IGF-I receptor has also been associated with preterm birth through a linkage analysis in the Finish population [39]. Interestingly, none of the remaining genes were previously implicated in preterm births. Their inclusion in this discovery sent was solely through our prior imputation [41]. The high degree of overlap of the same gene in different gene sets and the same genes in different modules is consistent with the well-recognized redundancy of genes and their networks in nature [47].

Whole exome sequencing (WES) has been undertaken to identify the genetic architecture of complex diseases [48–50]. While successful at identifying large numbers of variants, specificity is limited. A recent WES project from the National Heart, Lung and Blood Institute (NHLBI) identified almost 500,000 nucleotide variants which were rare [50]. Remarkably, individual patients were predicted to have up to 300 putatively deleterious variants but actual phenotype genotype correlations were not available. It was only after careful review of the literature and prioritization of the identified variants that targets for resequencing were identified [51, 52]. Our strategy is the inverse of the approach just described. We carried out the biological reductionism first through a robust literature curation and aggregation of genes from public databases [40]. We then used gene set enrichment with this biologically validated gene set to analyze a large genome wide association study of preterm birth [41]. We identified a modest number of genes and significant haplotype blocks. In this report we describe the results of targeted deep sequencing of these genes and significant haplotype blocks in women with a multigenerational history of preterm birth and compared the findings to patients delivering at term with no family history of preterm birth.

Our results are consistent with our *a priori* hypothesis that preterm birth would not be associated with single gene variant(s) but rather with variants in networks of genes. Additionally, we anticipated that we would find networks with gene variants in some but not all of the cases and controls. This is consistent with the notion that a minimal but sufficient disruption in several pathways is sufficient to lead to a clinical disease or phenotype but that different networks or modules can result in similar clinical or phenotypic outcomes [40, 53]. This approach is powerful at identifying subsets of patients with networks of genes that are associated with clinical disease phenotypes.

We compared cases with a multigenerational history of preterm birth to patients delivering at term with no family history of preterm birth. This approach is consistent with recommendations on design of studies to define rare variants and “missing heritability” which include careful phenotyping of cases, carefully-matched controls, use of *prior* data on genes or variants to identify targets and/or assess results [54, 55]. Our data are from a modest size cohort of patients. Nonetheless, our targeted strategy allowed us to find significant associations which both enhanced and reduced the risk of preterm birth. Other investigators have used combinations of targeted sequencing and/or targeted patient enrollment to enhance discovery of rare variants using modest patient size cohorts and have reported similar success [55–60]. As shown here, prior genetic analysis and prior filtering of both patients and gene targets improves the likelihood of identifying otherwise difficult-to-find rare variants [55–60]. Replication in another cohort of patients, comparison with genes associated evolutionarily with preterm birth and the addition of phylogenomic analyses are needed to validate and add veracity to these candidate genes [61].

These results illustrate an effective way to use large data sets and layered approaches employing pathway analysis, gene set enrichment and meta-genomic analysis, to identify the genes in networks and pathways associated with complex disease. We discovered modules of genes for which the variants in these genes taken together might prevent or result in preterm birth for a specified subset of patients. This meta-genomic approach is also suitable for meta-analysis of sequence/variant results from independent projects to identify gene networks in additional subsets of patients/phenotypes. These results are generalizable to other disorders.

## Methods

### Patient Identification and Enrollment

Women & Infants Hospital of Rhode Island is the only provider of high-risk perinatal services in Rhode Island, northeastern Connecticut and southeastern Massachusetts. We used this *population-based* service to enroll patients with a prior history of preterm birth. An informatically driven retrieval from our electronic medical record gave us a daily report on all preterm births. A clinical research assistant trained in genetic interviews reviewed the records of all patients delivering < 34 weeks. Controls were patients who delivered ≥ 37 weeks gestation in whom a careful, formal genetic history revealed no history of preterm birth on either maternal or paternal side of the pedigree. Following informed consent, women underwent a careful interview. There were explicit questions in the formal questionnaire of preterm birth in mother, grandmother, her first order relatives and also paternal relatives. Informed consent was obtained from all participants. Careful clinical history with an emphasis on additional risk factors for prematurity including medical illnesses, drug use, psychiatric disorders and employment history was recorded on all patients. The study was approved by the Institutional Review Board, 08–0117. 48 samples were selected for targeted sequencing. Samples were taken from 23 women with 2 generations of preterm birth, 9 women with 3 generations of preterm birth and 16 control women at term. The clinical characteristics of the patients are shown in Table 4. The only significant difference was the older gestational age among the controls. All of the patients’ identifying data was coded and redacted for the purposes of data analysis. Residual maternal whole blood was obtained for extraction of genomic DNA. The samples were stored continuously at -80°C until processing.

**Table 4:** Clinical characteristics of maternal patients (mean ± SD).

Study Group	Maternal Age	Gravida	Gestational Age	Race
Preterm	25.0 ± 5.3	2.8 ± 2.3	31.9 ± 2.3	A8; AS1; H8; W18;NA1
Controls	24.8 ± 4.5	1.8 ± 1	40.0 ± 0.7*	A3; AS1; H3; W9

* P<0.05A;

A African-American; AS Asian; H Hispanic; W White; NA Native American

### Sample preparation

We targeted 329 genes and 132 haplotype blocks that are highly associated with preterm birth for sequencing [41]. Genomic DNA from whole blood was extracted using DNA kit QIAamp DSP DNA blood mini kit from Qiagen following the manufacture’s protocol. Samples were quantified using Qubit technology (Life Technologies, Carlsbad, CA, USA) and sequencing libraries were constructed from 2 **μ**g each of case/control DNA. Library preparation was performed using Illumina TruSeq DNA LT Sample prep Kit (Illumina, San Diego, CA, USA), with enzymatic fragmentation using ds DNA Fragmentase (NEB), followed by indexing and clean-up. DNA capture was performed using custom capture probes from SeqCap EZ choice kit (Roche NimbleGen) Post-capture quality control and targeted sequencing were performed at the Brown University Genomics Core.

### Targeted sequencing

The library was sequenced on our Illumina *HiSeq* 2500 using 100 base pair paired-end protocols. Initial cluster counts of ~300,000 were obtained. Following sequencing, the multiplex indices were used to bin the samples for each patient and QC sequence data was recorded. High quality sequence data from well-balanced pools was observed. There were an average of 22,000,000 reads from each patient, with an average of 99% perfect index reads and a Q30 of 91%. The mean Phred score for each patient was 36. These data were then aligned to the human reference sequence (Hg19). Reads were mapped to the to the human reference sequence (Hg19) with BWA [62] sorted and indexed with SAMtools [63].

### Sequence data, variant calling and zygosity testing

Variants were flagged as low quality and filtered using the established metrics: if three or more variants detected within 10bp; if four or more alignments map to different locations equally well; if coverage of less than five reads; if quality score < 30; if low quality for a particular sequence depth (variant confidence/unfiltered depth < 1.5); and if strand bias (Phred-scaled p-values using Fisher’s Exact Test > 200). A variant identified by any ONE of these filters was labeled “low quality” and not considered for further analysis. For variant discovery we used the Gene Analysis Tool Kit (GATK) version 3.2 to analyze the sequence reads. Following the filters described in Methods, we implemented GATK’s *Haplotype Caller* [64, 65]. Duplicated reads marked and removed using Picard Tools version 1.77. Haplotype caller was applied for variant detection on 329 gene set library and the Haplotype blocks library. 100 base pairs upstream and downstream of the each gene were included in the variant detection.

### Annotations

Variants were annotated using ANNOVAR for functional prediction scores Polyphen 2 HDIV [prediction if a change is damaging (> = 0.957), possibly damaging (0.453< = Polyphen 2 HDIV < = 0.956) or benign < = 0.452], PhyloP [prediction of a conserved (>0.95) or non- conserved (<0.95) site], and chromosome position [66].

### Univariate Analysis

In order to focus on putatively relevant variants, we eliminated the bulk of the identified variants for immediate investigation because they were equally common in cases and controls. Using a Markov Chain Monte Carlo (MCMC) Fisher Exact Test, we created a 2 x 3 contingency table for zygosity testing to compare the frequency of homozygosity for the reference allele, heterozygosity or homozygosity for the minor allele. The results are shown in Table 3.

### Meta-Analysis using GSEA and iBBiG

For each patient (total of 48), we built a gene list for each patient for GSEA. In order to ensure that we analyzed enough genes in each patient to conduct gene set enrichment, we relaxed the significance threshold on genes from p <.05 to p <.1. We ran GSEA on each patient’s gene list independently [43]. We used pre-ranked GSEA analysis against a collection of curated gene sets (C2) from MSigDB [43]. The resultant gene sets were considered significant if they obtained a nominal p-value below 0.05. Next, we transformed the significant gene sets into a binary matrix where the rows of the matrix were the gene sets and the columns were the individual patients. The binary matrix was used as input into the iterative binary bi-clustering algorithm (iBBiG). This algorithm was used to identify groups of gene sets that are coordinately associated with subsets of patients and their phenotypes (preterm, term) across the GSEA results. Fisher exact test was used to compare the abundance of cases and controls in each module output from iBBiG.

### Gene Ontology

We sought to provide a representation of the biological processes encompassed by these gene sets using the Gene Ontology (GO) Database. The Gene Ontology Database describes genes in terms of their associated biological processes, molecular functions and cellular components. Using GOstat, the genes shown in the clusters were tested for their statistical association with GO terms [67]. The program identifies Gene Ontology terms for which genes in the list were overrepresented. For each GO term, a p*-*value was calculated indicating the probability that the observed counts could have resulted from randomly distributing the associated GO terms between our genes and modules. GOstat corrects for multiple comparisons by employing a false discovery rate, *p* <.05.

## Supporting Information

S1 Table Genes and variants from zygosity testing (Exome library). (DOCX)

S2 Table Genes and variants from zygosity testing (Haplotype library). (DOCX)

S3 Table Gene ontology terms from each significant module. Genes from each module were used in GO to describe biological functions.16 Each biological function and module number is shown for the nine clusters. Modules are shown as large solid figures. Connections (“edges”) between biological functions in different clusters are shown.
(DOCX)
