# Supplementary material for: Targeted Sequencing and Meta-Analysis of Preterm Birth
Source: PLoS One. 2016 May 10;11(5):e0155021. doi: 10.1371/journal.pone.0155021 (PMC4862658; doi:10.1371/journal.pone.0155021)
Supplement: S1 Table — (DOCX) [file pone.0155021.s001.docx]

**S1 Table.** Variants only for Exome library, p<0.05 according to MCMC calculations.

| **Gene** | **HGNC ID** | **Chr** | **Start** | **Function** |
| --- | --- | --- | --- | --- |
| ARHGEF10L | 25540 | 1 | 17929990 | intronic |
| COL16A1 | 2193 | 1 | 32149263 | intronic |
| COL16A1 | 2193 | 1 | 32154650 | splicing |
| FAF1 | 3578 | 1 | 50956163 | intronic |
| PTGFR | 9600 | 1 | 78958491 | exonic |
| PGLYRP3 | 30014 | 1 | 153279462 | intronic |
| SPTA1 | 11272 | 1 | 158582646 | exonic |
| SPTA1 | 11272 | 1 | 158647522 | exonic |
| SPTA1 | 11272 | 1 | 158647669 | intronic |
| SPTA1 | 11272 | 1 | 158654852 | intronic |
| ATF6 | 791 | 1 | 161823197 | intronic |
| ATF6 | 791 | 1 | 161929235 | UTR3 |
| ATF6 | 791 | 1 | 161931562 | UTR3 |
| ATF6 | 791 | 1 | 161932193 | UTR3 |
| SOAT1 | 11177 | 1 | 179324008 | 3_prime_UTR_variant |
| SOAT1 | 11177 | 1 | 179326052 | UTR3 |
| SOAT1 | 11177 | 1 | 179326054 | UTR3 |
| SOAT1 | 11177 | 1 | 179327327 | UTR3 |
| PTGS2 | 9605 | 1 | 186647323 | intronic |
| PLA2G4A | 9035 | 1 | 186798045 | 5_prime_UTR_variant |
| USH2A | 12601 | 1 | 215931901 | intronic |
| USH2A | 12601 | 1 | 216258213 | exonic |
| USH2A | 12601 | 1 | 216270618 | intronic |
| ALK | 427 | 2 | 29455267 | exonic |
| ORC4 | 8490 | 2 | 148688922 | UTR3 |
| ORC4 | 8490 | 2 | 148689494 | UTR3 |
| ORC4 | 8490 | 2 | 148778340 | UTR5 |
| UBR3 | 30467 | 2 | 170782356 | intronic |
| UBR3 | 30467 | 2 | 170783765 | intronic |
| UBR3 | 30467 | 2 | 170863471 | intronic |
| UBR3 | 30467 | 2 | 170939692 | UTR3 |
| GAD1 | 4092 | 2 | 171700729 | intronic |
| GAD1 | 4092 | 2 | 171713564 | exonic |
| NCKAP1 | 7666 | 2 | 183789488 | downstream |
| NCKAP1 | 7666 | 2 | 183903523 | UTR5 |
| ERBB4 | 3432 | 2 | 212242192 | UTR3 |
| ERBB4 | 3432 | 2 | 212285103 | intronic |
| ERBB4 | 3432 | 2 | 212488632 | intronic |
| ERBB4 | 3432 | 2 | 212522450 | intronic |
| ERBB4 | 3432 | 2 | 212522651 | intronic |
| GOLGA4 | 4427 | 3 | 37284822 | UTR5 |
| GOLGA4 | 4427 | 3 | 37284829 | 5_prime_UTR_variant |
| DZIP3 | 30938 | 3 | 108324365 | intronic |
| DZIP3 | 30938 | 3 | 108366978 | intronic |
| DZIP3 | 30938 | 3 | 108373022 | exonic |
| DZIP3 | 30938 | 3 | 108394771 | intronic |
| DZIP3 | 30938 | 3 | 108413438 | UTR3 |
| DZIP3 | 30938 | 3 | 108413441 | UTR3 |
| GSK3B | 4617 | 3 | 119540967 | UTR3 |
| GSK3B | 4617 | 3 | 119544615 | UTR3 |
| MYLK-AS1 | 42440 | 3 | 123332186 | ncRNA_intronic |
| MYLK | 7590 | 3 | 123368043 | splicing |
| MYLK | 7590 | 3 | 123376067 | exonic |
| MGLL | 17038 | 3 | 127408182 | UTR3 |
| MGLL | 17038 | 3 | 127408239 | UTR3 |
| MGLL | 17038 | 3 | 127410915 | UTR3 |
| ATP2C1 | 13211 | 3 | 130660392 | intronic |
| CHST2 | 1970 | 3 | 142841740 | UTR3 |
| SERPINI1 | 8943 | 3 | 167540741 | intronic |
| PLD1 | 9067 | 3 | 171405424 | intronic |
| NLGN1 | 14291 | 3 | 173152748 | intronic |
| NLGN1 | 14291 | 3 | 173152783 | intronic |
| DLG1 | 2900 | 3 | 196778438 | intronic |
| ANAPC4 | 19990 | 4 | 25379226 | intronic |
| ANAPC4 | 19990 | 4 | 25396001 | intronic |
| CCNG2 | 1593 | 4 | 78089831 | 3_prime_UTR_variant |
| LOC100507053 | - | 4 | 100131746 | ncRNA_intronic |
| ADH1C | 251 | 4 | 100265957 | intronic |
| ADH7 | 256 | 4 | 100350594 | intronic |
| ADH7 | 256 | 4 | 100356466 | UTR5 |
| EDNRA | 3179 | 4 | 148457005 | intronic |
| IL31RA | 18969 | 5 | 55185987 | intronic |
| F2RL2 | 3539 | 5 | 75911399 | UTR3 |
| F2RL2 | 3539 | 5 | 75911464 | UTR3 |
| IQGAP2 | 6111 | 5 | 75911568 | intronic |
| F2RL2 | 3539 | 5 | 75911715 | UTR3 |
| F2RL2 | 3539 | 5 | 75911987 | UTR3 |
| F2RL2 | 3539 | 5 | 75912401 | UTR3 |
| F2RL2 | 3539 | 5 | 75912843 | UTR3 |
| IQGAP2 | 3539 | 5 | 75914517 | intronic |
| IQGAP2 | 3539 | 5 | 75954537 | intronic |
| IQGAP2 | 3539 | 5 | 75954577 | intronic |
| IQGAP2 | 3539 | 5 | 75967862 | intronic |
| IQGAP2 | 3539 | 5 | 75969747 | intronic |
| IQGAP2 | 3539 | 5 | 75973031 | intronic |
| IQGAP2 | 3539 | 5 | 75996865 | intronic |
| ADGRV1 | 17416 | 5 | 89925388 | intronic |
| ADGRV1 | 17416 | 5 | 90445889 | exonic |
| ADGRV1 | 17416 | 5 | 90459777 | UTR3 |
| TNFAIP8 | 17260 | 5 | 118691737 | UTR5 |
| TNFAIP8 | 17260 | 5 | 118691832 | intronic |
| ALDH7A1 | 877 | 5 | 125879853 | UTR3 |
| ALDH7A1 | 877 | 5 | 125879855 | UTR3 |
| MAPK14 | 6876 | 6 | 36040642 | intronic |
| MAPK14 | 6876 | 6 | 36078180 | UTR3 |
| TAF8 | 17300 | 6 | 42045689 | 3_prime_UTR_variant |
| IL6 | 6018 | 7 | 22768249 | intronic |
| HNRNPA2B1 | 5033 | 7 | 26235413 | intronic |
| HNRNPA2B1 | 5033 | 7 | 26240298 | UTR5 |
| AOAH | 548 | 7 | 36660502 | intronic |
| AOAH | 548 | 7 | 36698684 | intronic |
| HUS1 | 5309 | 7 | 48003360 | UTR3 |
| ADHFE1 | 16354 | 8 | 67344690 | upstream |
| ADHFE1 | 16354 | 8 | 67357063 | intronic |
| ADHFE1 | 16354 | 8 | 67357071 | intronic |
| IFNWP9 | 5455 | 9 | 21186596 | downstream |
| IFNA4 | 5425 | 9 | 21186931 | UTR3 |
| IFNA4 | 5425 | 9 | 21187121 | exonic |
| TSC1 | 12362 | 9 | 135769064 | UTR3 |
| PARD3 | 16051 | 10 | 34666864 | intronic |
| ARRB1 | 711 | 11 | 74982794 | intronic |
| PAK1 | 8590 | 11 | 77051820 | intronic |
| ATM | 795 | 11 | 108236513 | UTR3 |
| ATM | 795 | 11 | 108237839 | UTR3 |
| ATM | 795 | 11 | 108238010 | UTR3 |
| ATM | 795 | 11 | 108238991 | UTR3 |
| ATM | 795 | 11 | 108239628 | UTR3 |
| ZBTB16 | 12930 | 11 | 114120999 | intronic |
| ETS1 | 3488 | 11 | 128350380 | intronic |
| BCAT1 | 976 | 12 | 24967033 | UTR3 |
| BCAT1 | 976 | 12 | 24968749 | UTR3 |
| CSAD | 18966 | 12 | 53551384 | downstream |
| CSAD | 18966 | 12 | 53551387 | downstream |
| CSAD | 18966 | 12 | 53551860 | UTR3 |
| IGF1 | 5464 | 12 | 102790780 | UTR3 |
| STAB2 | 18629 | 12 | 104152856 | intronic |
| STAB2 | 18629 | 12 | 104153004 | exonic |
| TBX5 | 11604 | 12 | 114845607 | intronic |
| TBX5 | 11604 | 12 | 114845885 | UTR5 |
| TBX5-AS1 | 27402 | 12 | 114846228 | ncRNA_intronic |
| NOS1 | 7872 | 12 | 117646227 | UTR3 |
| NOS1 | 7872 | 12 | 117647983 | UTR3 |
| NOS1 | 7872 | 12 | 117647985 | UTR3 |
| NOS1 | 7872 | 12 | 117647986 | UTR3 |
| NOS1 | 7872 | 12 | 117648892 | UTR3 |
| NOS1 | 7872 | 12 | 117648967 | UTR3 |
| NOS1 | 7872 | 12 | 117648982 | UTR3 |
| NOS1 | 7872 | 12 | 117649004 | UTR3 |
| NOS1 | 7872 | 12 | 117649008 | UTR3 |
| NOS1 | 7872 | 12 | 117649015 | UTR3 |
| NOS1 | 7872 | 12 | 117649825 | UTR3 |
| NOS1 | 7872 | 12 | 117652057 | UTR3 |
| TAOK3 | 18133 | 12 | 118587505 | downstream |
| WASF3 | 12734 | 13 | 27257004 | exonic |
| FLT1 | 3763 | 13 | 28893484 | intronic |
| NBEA | 7648 | 13 | 35806549 | intronic |
| IRS2 | 6126 | 13 | 110406869 | UTR3 |
| IRS2 | 6126 | 13 | 110408608 | UTR3 |
| CDC16 | 1720 | 13 | 115002097 | intronic |
| PLCB2 | 9055 | 15 | 40582767 | intronic |
| CYP19A1 | 2594 | 15 | 51535192 | intronic |
| AQP9 | 643 | 15 | 58465171 | intronic |
| AQP9 | 643 | 15 | 58465483 | intronic |
| AQP9 | 643 | 15 | 58471368 | exonic |
| AQP9 | 643 | 15 | 58476599 | UTR3 |
| AQP9 | 643 | 15 | 58476738 | UTR3 |
| AQP9 | 643 | 15 | 58477363 | 3_prime_UTR_variant |
| AQP9 | 643 | 15 | 58477797 | UTR3 |
| ANXA2 | 537 | 15 | 60690089 | exonic |
| SLC28A1 | 11001 | 15 | 85448906 | intronic |
| ISG20 | 6130 | 15 | 89179531 | intronic |
| ISG20 | 6130 | 15 | 89179555 | intronic |
| IQGAP1 | 6110 | 15 | 90999351 | intronic |
| IQGAP1 | 6110 | 15 | 91009484 | intronic |
| CIITA | 7067 | 16 | 11018249 | UTR3 |
| WWP2 | 16804 | 16 | 69967969 | exonic |
| SMG6 | 17809 | 17 | 1963961 | UTR3 |
| SLC2A4 | 11009 | 17 | 7190629 | UTR3 |
| TP53 | 11998 | 17 | 7571985 | UTR3 |
| CDC6 | 1744 | 17 | 38451739 | intronic |
| COX11 | 2261 | 17 | 53039325 | 3_prime_UTR_variant |
| RNFT1 | 30206 | 17 | 58026021 | 3_prime_UTR_variant |
| PRPSAP1 | 9466 | 17 | 74349910 | UTR5 |
| RALBP1 | 9841 | 18 | 9531034 | intronic |
| SERPINB8 | 8952 | 18 | 61654463 | exonic |
| AZU1 | 913 | 19 | 830820 | exonic |
| ANGPTL4 | 16039 | 19 | 8429066 | UTR5 |
| MAP4K1 | 6863 | 19 | 39106797 | intronic |
| PSG1 | 9514 | 19 | 43383680 | exonic |
| RUVBL2 | 10475 | 19 | 49513273 | exonic |
| RUVBL2 | 10475 | 19 | 49513736 | intronic |
| LHB,RUVBL2 | 6584 | 19 | 49519230 | downstream |
| MYH14 | 23212 | 19 | 50764642 | intronic |
| MYH14 | 23212 | 19 | 50766707 | intronic |
| SLC23A2 | 10973 | 20 | 4834894 | UTR3 |
| SLC23A2 | 10973 | 20 | 4990727 | intronic |
| PTGIS | 9603 | 20 | 48123142 | UTR3 |
| PTGIS | 9603 | 20 | 48123145 | UTR3 |
| PTGIS | 9603 | 20 | 48123162 | UTR3 |
| APP | 620 | 21 | 27327100 | intronic |
| APP | 620 | 21 | 27348372 | intronic |
| NF2 | 7773 | 22 | 29999878 | UTR5 |
| MYH9 | 7579 | 22 | 36684980 | exonic |
| APOBEC3F | 17356 | 22 | 39450439 | UTR3 |
| APOBEC3F | 17356 | 22 | 39450443 | UTR3 |
| APOBEC3F | 17356 | 22 | 39451432 | UTR3 |
| APOBEC3F | 17356 | 22 | 39451441 | UTR3 |
| APOBEC3F | 17356 | 22 | 39451443 | UTR3 |
| PNPLA3 | 18590 | 22 | 44323123 | intronic |
| PNPLA3 | 18590 | 22 | 44323129 | intronic |
| PNPLA3 | 18590 | 22 | 44323147 | intronic |
| PNPLA3 | 18590 | 22 | 44342116 | exonic |
| SHROOM2 | 630 | X | 9915849 | UTR3 |
| TLR7 | 15631 | X | 12906714 | exonic |
| SLC9A7 | 17123 | X | 46464526 | UTR3 |
