# Supplementary material for: Targeted Sequencing and Meta-Analysis of Preterm Birth
Source: PLoS One. 2016 May 10;11(5):e0155021. doi: 10.1371/journal.pone.0155021 (PMC4862658; doi:10.1371/journal.pone.0155021)
Supplement: S2 Table — (DOCX) [file pone.0155021.s002.docx]

**S2 Table.** Variants only for Haplotype block library, p<0.05 according to MCMC calculations.

| **Gene** | **HGNC ID** | **Chr** | **Pos** | **Function** |
| --- | --- | --- | --- | --- |
| WASF2 | 12733 | 1 | 27769840 | intronic |
| COL16A1 | 2193 | 1 | 32135022 | intronic |
| COL16A1 | 2193 | 1 | 32136995 | intronic |
| COL16A1 | 2193 | 1 | 32137510 | intronic |
| AP4B1-AS1 | 44114 | 1 | 114443343 | ncRNA_exonic |
| NOTCH2 | 7882 | 1 | 120544333 | intronic |
| PGLYRP3 | 30014 | 1 | 153279274 | intronic |
| IL6R | 6019 | 1 | 154409755 | intronic |
| IL6R | 6019 | 1 | 154419843 | intronic |
| SPTA1 | 11272 | 1 | 158620647 | intronic |
| SOAT1 | 11177 | 1 | 179305368 | intronic |
| AKT3 | 393 | 1 | 243779250 | intronic |
| AKT3 | 393 | 1 | 243808483 | intronic |
| AKT3 | 393 | 1 | 243855995 | intronic |
| AKT3 | 393 | 1 | 244000200 | intronic |
| AKT3 | 393 | 1 | 244003535 | intronic |
| SOS1 | 11187 | 2 | 39265603 | intronic |
| SOS1 | 11187 | 2 | 39265605 | intronic |
| SOS1 | 11187 | 2 | 39265614 | intronic |
| SOS1 | 11187 | 2 | 39281312 | intronic |
| SOS1 | 11187 | 2 | 39285372 | intronic |
| ACTR2 | 169 | 2 | 65478657 | intronic |
| XRCC5 | 12833 | 2 | 217049473 | intronic |
| SAG | 10521 | 2 | 234234000 | intronic |
| SAG | 10521 | 2 | 234234922 | intronic |
| SAG | 10521 | 2 | 234237387 | intronic |
| SAG | 10521 | 2 | 234243322 | intronic |
| PPARG | 9236 | 3 | 12423111 | intronic |
| PPARG | 9236 | 3 | 12424400 | intronic |
| PPARG | 9236 | 3 | 12424415 | intronic |
| PPARG | 9236 | 3 | 12424420 | intronic |
| PPARG | 9236 | 3 | 12424423 | intronic |
| PPARG | 9236 | 3 | 12429300 | intronic |
| PPARG | 9236 | 3 | 12431273 | intronic |
| PPARG | 9236 | 3 | 12431307 | intronic |
| PPARG | 9236 | 3 | 12431319 | intronic |
| PPARG | 9236 | 3 | 12431323 | intronic |
| PPARG | 9236 | 3 | 12455118 | intronic |
| PPARG | 9236 | 3 | 12455119 | intronic |
| PPARG | 9236 | 3 | 12455141 | intronic |
| RAF1 | 9829 | 3 | 12634414 | intronic |
| RAF1 | 9829 | 3 | 12637844 | intronic |
| RAF1 | 9829 | 3 | 12637951 | intronic |
| RAF1 | 9829 | 3 | 12639152 | intronic |
| RAF1 | 9829 | 3 | 12639202 | intronic |
| RAF1 | 9829 | 3 | 12639203 | intronic |
| RAF1 | 9829 | 3 | 12639205 | intronic |
| RAF1 | 9829 | 3 | 12639715 | intronic |
| RAF1 | 9829 | 3 | 12639718 | intronic |
| RAF1 | 9829 | 3 | 12639721 | intronic |
| RAF1 | 9829 | 3 | 12648917 | intronic |
| RAF1 | 9829 | 3 | 12653177 | intronic |
| RAF1 | 9829 | 3 | 12660739 | intronic |
| RAF1 | 9829 | 3 | 12661955 | intronic |
| RAF1 | 9829 | 3 | 12666402 | intronic |
| RAF1 | 9829 | 3 | 12671510 | intronic |
| RAF1 | 9829 | 3 | 12671550 | intronic |
| RAF1 | 9829 | 3 | 12671564 | intronic |
| RAF1 | 9829 | 3 | 12675583 | intronic |
| RAF1 | 9829 | 3 | 12683952 | intronic |
| RAF1 | 9829 | 3 | 12685243 | intronic |
| RAF1 | 9829 | 3 | 12690527 | intronic |
| SH3BP5-AS1 | 44501 | 3 | 15303992 | ncRNA_intronic |
| PLA1A | 17661 | 3 | 119339054 | intronic |
| SERPINI1 | 8943 | 3 | 167535625 | intronic |
| TNFAIP8 | 17260 | 5 | 118724562 | intronic |
| TNFAIP8 | 17260 | 5 | 118726637 | intronic |
| TNFAIP8 | 17260 | 5 | 118727592 | intronic |
| MAPK14 | 6876 | 6 | 35998388 | intronic |
| MAPK14 | 6876 | 6 | 36013437 | intronic |
| MAPK14 | 6876 | 6 | 36013459 | intronic |
| MAPK14 | 6876 | 6 | 36065664 | intronic |
| LIMK1 | 6613 | 7 | 73522795 | intronic |
| LIMK1 | 6613 | 7 | 73522796 | intronic |
| PIK3CG | 8978 | 7 | 106516651 | intronic |
| PIK3CG | 8978 | 7 | 106520605 | intronic |
| ADHFE1 | 16354 | 8 | 67367114 | intronic |
| ADHFE1 | 16354 | 8 | 67371796 | intronic |
| AMBP | 453 | 9 | 116824255 | intronic |
| TSC1 | 12362 | 9 | 135785513 | intronic |
| SORBS1 | 14565 | 10 | 97080325 | intronic |
| SORBS1 | 14565 | 10 | 97080415 | intronic |
| CHUK | 1974 | 10 | 101956301 | intronic |
| CHUK | 1974 | 10 | 101956302 | intronic |
| CHUK | 1974 | 10 | 101956305 | intronic |
| CHUK | 1974 | 10 | 101956306 | intronic |
| CHUK | 1974 | 10 | 101966084 | intronic |
| CHUK | 1974 | 10 | 101966098 | intronic |
| CHUK | 1974 | 10 | 101968114 | intronic |
| CHUK | 1974 | 10 | 101968403 | intronic |
| CHUK | 1974 | 10 | 101968405 | intronic |
| CHUK | 1974 | 10 | 101973012 | intronic |
| CHUK | 1974 | 10 | 101981670 | intronic |
| CHUK | 1974 | 10 | 101981688 | intronic |
| SPI1 | 11241 | 11 | 47397005 | intronic |
| PAK1 | 8590 | 11 | 77055232 | intronic |
| PAK1 | 8590 | 11 | 77055235 | intronic |
| PAK1 | 8590 | 11 | 77060528 | intronic |
| PAK1 | 8590 | 11 | 77069804 | intronic |
| PAK1 | 8590 | 11 | 77091141 | intronic |
| PGR | 8910 | 11 | 100915713 | intronic |
| PGR | 8910 | 11 | 100934955 | intronic |
| PGR | 8910 | 11 | 100934956 | intronic |
| ETS1 | 3488 | 11 | 128391937 | UTR5 |
| ETNK1 | 24649 | 12 | 22801281 | intronic |
| ETNK1 | 24649 | 12 | 22823667 | intronic |
| ETNK1 | 24649 | 12 | 22826751 | intronic |
| BCAT1 | 976 | 12 | 24980519 | intronic |
| BCAT1 | 976 | 12 | 25014396 | intronic |
| BCAT1 | 976 | 12 | 25030854 | intronic |
| KITLG | 6343 | 12 | 88925973 | intronic |
| KITLG | 6343 | 12 | 88939133 | intronic |
| NOS1 | 7872 | 12 | 117685750 | intronic |
| TAOK3 | 18133 | 12 | 118615594 | intronic |
| TAOK3 | 18133 | 12 | 118682492 | intronic |
| TAOK3 | 18133 | 12 | 118693769 | intronic |
| TAOK3 | 18133 | 12 | 118702566 | intronic |
| TAOK3 | 18133 | 12 | 118702570 | intronic |
| TAOK3 | 18133 | 12 | 118776971 | intronic |
| ATP7B | 870 | 13 | 52515691 | intronic |
| ATP7B | 870 | 13 | 52515692 | intronic |
| ATP7B | 870 | 13 | 52536266 | intronic |
| MBIP | 20427 | 14 | 36786343 | intronic |
| RDH12 | 19977 | 14 | 68175946 | intronic |
| RDH12 | 19977 | 14 | 68182224 | intronic |
| RDH12 | 19977 | 14 | 68189736 | intronic |
| RDH12 | 19977 | 14 | 68192653 | intronic |
| SERPINA4 | 8948 | 14 | 95034342 | intronic |
| AKT1 | 391 | 14 | 105240606 | intronic |
| AKT1 | 391 | 14 | 105243623 | intronic |
| IQGAP1 | 6110 | 15 | 90992687 | intronic |
| SMG1 | 30045 | 16 | 18847071 | intronic |
| SMG1 | 30045 | 16 | 18856299 | intronic |
| SMG1 | 30045 | 16 | 18862729 | intronic |
| SMG1 | 30045 | 16 | 18862741 | intronic |
| SMG6 | 17809 | 17 | 1965085 | intronic |
| SMG6 | 17809 | 17 | 1965096 | intronic |
| SMG6 | 17809 | 17 | 1976692 | intronic |
| SMG6 | 17809 | 17 | 1976713 | intronic |
| SMG6 | 17809 | 17 | 1976716 | intronic |
| SMG6 | 17809 | 17 | 1976925 | intronic |
| SMG6 | 17809 | 17 | 1976926 | intronic |
| SMG6 | 17809 | 17 | 1976927 | intronic |
| TOM1L1 | 11983 | 17 | 53001829 | intronic |
| TOM1L1 | 11983 | 17 | 53009971 | intronic |
| TOM1L1 | 11983 | 17 | 53037589 | intronic |
| TOM1L1 | 11983 | 17 | 53037592 | intronic |
| TOM1L1 | 11983 | 17 | 53037598 | intronic |
| RPS6KB1 | 10436 | 17 | 57989860 | intronic |
| RPS6KB1 | 10436 | 17 | 57992410 | intronic |
| RPS6KB1 | 10436 | 17 | 58014205 | intronic |
| RPS6KB1 | 10436 | 17 | 58014277 | intronic |
| RPS6KB1 | 10436 | 17 | 58020343 | intronic |
| PRPSAP1 | 9466 | 17 | 74309474 | intronic |
| RALBP1 | 9841 | 18 | 9523967 | intronic |
| ANGPTL4 | 16039 | 19 | 8435082 | intronic |
| ANGPTL4 | 16039 | 19 | 8435585 | intronic |
| MAP4K1 | 6863 | 19 | 39088702 | intronic |
| BCL2L1 | 992 | 20 | 30290192 | intronic |
| PTGIS | 9603 | 20 | 48126879 | intronic |
| NF2 | 7773 | 22 | 30033036 | intronic |
| NF2 | 7773 | 22 | 30061326 | intronic |
| NF2 | 7773 | 22 | 30068066 | intronic |
| NF2 | 7773 | 22 | 30075845 | intronic |
| NF2 | 7773 | 22 | 30075850 | intronic |
| MYH9 | 7579 | 22 | 36720851 | intronic |
| MYH9 | 7579 | 22 | 36745749 | intronic |
| PLA2G6 | 9039 | 22 | 38566331 | intronic |
