# Supplementary material for: Targeted Sequencing and Meta-Analysis of Preterm Birth
Source: PLoS One. 2016 May 10;11(5):e0155021. doi: 10.1371/journal.pone.0155021 (PMC4862658; doi:10.1371/journal.pone.0155021)
Supplement: S3 Table — Genes from each module were used in GO to describe biological functions.16 Each biological function and module number is shown for the nine clusters. Modules are shown as large solid figures. Connections (“edges”) between biological functions in different clusters are shown. (DOCX) [file pone.0155021.s003.docx]

**S3 Table.** GO terms and significant modules.

| **Groups** | **GO Terms** | **Modules** |
| --- | --- | --- |
| A | behavior | HM8 |
| A | cell migration | HM8 |
| A | cell motility | HM8 |
| A | chemotaxis | HM8 |
| A | localization of cell | HM8 |
| A | locomotory behavior | HM8 |
| A | positive regulation of cell migration | HM8 |
| A | positive regulation of cell motion | HM8 |
| A | positive regulation of locomotion | HM8 |
| A | positive regulation of smooth muscle cell proliferation | HM8 |
| A | regulation of smooth muscle cell proliferation | HM8 |
| A | response to bacterium | HM8 |
| A | response to corticosteroid stimulus | HM8 |
| A | response to ethanol | HM8 |
| A | response to glucocorticoid stimulus | HM8 |
| A | response to lipopolysaccharide | HM8 |
| A | response to molecule of bacterial origin | HM8 |
| A | taxis | HM8 |
| B | regulation of cell motion | HM8, HM9 |
| B | response to steroid hormone stimulus | HM8, HM9 |
| B | regulation of protein amino acid phosphorylation | HM8, HM9 |
| B | regulation of protein modification process | HM8, HM9 |
| B | regulation of MAPKKK cascade | HM8, HM9 |
| B | regulation of cell migration | HM8, HM9 |
| B | regulation of tyrosine phosphorylation of STAT protein | HM8, HM9 |
| B | regulation of protein kinase cascade | HM8, HM9 |
| B | regulation of tyrosine phosphorylation of Stat3 protein | HM8, HM9 |
| B | regulation of cell proliferation | HM8, HM9 |
| B | response to peptide hormone stimulus | HM8, HM9 |
| B | regulation of locomotion | HM8, HM9 |
| B | regulation of JAK-STAT cascade | HM8, HM9 |
| C | positive regulation of developmental process | HM8, HM9,HM2 |
| C | regulation of peptidyl-tyrosine phosphorylation | HM8, HM9,HM2 |
| D | regulation of response to external stimulus | HM9 |
| D | negative regulation of multicellular organismal process | HM9 |
| D | negative regulation of macromolecule metabolic process | HM9 |
| D | regulation of cytokine production | HM9 |
| D | negative regulation of biosynthetic process | HM9 |
| D | negative regulation of macromolecule biosynthetic process | HM9 |
| D | negative regulation of catalytic activity | HM9 |
| D | regulation of phosphorus metabolic process | HM9 |
| D | regulation of transferase activity | HM9 |
| D | regulation of phosphate metabolic process | HM9 |
| D | regulation of phosphorylation | HM9 |
| D | negative regulation of transferase activity | HM9 |
| D | regulation of cellular protein metabolic process | HM9 |
| E | negative regulation of cytokine production | HM9, HM2 |
| E | positive regulation of cell differentiation | HM9, HM2 |
| F | protein amino acid phosphorylation | HM2 |
| F | positive regulation of neurogenesis | HM2 |
| F | positive regulation of signal transduction | HM2 |
| F | positive regulation of cell development | HM2 |
| F | phosphorus metabolic process | HM2 |
| F | phosphorylation | HM2 |
| F | regulation of anti-apoptosis | HM2 |
| F | positive regulation of MAPKKK cascade | HM2 |
| F | nerve growth factor receptor signaling pathway | HM2 |
| F | positive regulation of cell communication | HM2 |
| F | positive regulation of macromolecule metabolic process | HM2 |
| F | phosphate metabolic process | HM2 |
| F | immune system development | HM2 |
| F | lipid homeostasis | HM2 |
| F | Ras protein signal transduction | HM2 |
| F | hemopoietic or lymphoid organ development | HM2 |
| F | death | HM2 |
| F | positive regulation of peptidyl-tyrosine phosphorylation | HM2 |
| F | hemopoiesis | HM2 |
| F | cell death | HM2 |
| F | myeloid leukocyte differentiation | HM2 |
| G | extracellular structure organization | EM4 |
| G | extracellular matrix organization | EM4 |
| H | intracellular signaling cascade | HM2, EM8 |
| H | small GTPase mediated signal transduction | HM2, EM8 |
| I | regulation of small GTPase mediated signal transduction | EM8 |
| I | regulation of mitotic cell cycle | EM8 |
| I | regulation of cell cycle process | EM8 |
| I | regulation of nuclear division | EM8 |
| I | positive regulation of protein kinase cascade | EM8 |
| I | regulation of mitosis | EM8 |
